# Comparison of the operative time for open door laminoplasty using titanium plate spacers or hydroxyapatite block spacers: a retrospective study

**DOI:** 10.1186/s13018-019-1539-5

**Published:** 2019-12-30

**Authors:** Takahiro Inui, Koichi Inokuchi, Yoshinobu Watanabe, Kentaro Matsui, Yuhei Nakayama, Keisuke Ishii, Takashi Suzuki, Taketo Kurozumi, Hirotaka Kawano

**Affiliations:** 1 Department of Orthopaedic Surgery, Teikyo University School of Medicine, Tokyo, Japan; 2 Trauma and Reconstruction Center, Teikyo University Hospital, Tokyo, Japan; 3 Department of Emergency Medicine, Saitama Medical Center, Saitama Medical University, Saitama, Japan

**Keywords:** Open door laminoplasty, Laminoplasty, Operation time, Hydroxyapatite, Titanium plate, Retrospective, Comparative study, Spinal cord injury without bone injury

## Abstract

**Abstract:**

**Background:**

Titanium plate (TP) and hydroxyapatite (HA) spacers are widely used during open-door laminoplasty, performed with the patient in a prone position. Reducing operative time is an important consideration, particularly to reduce the risk of postoperative complications in older patients. The purpose of this retrospective cohort study was to compare the operative time for open-door laminoplasty using TP or HA spacers.

**Methods:**

Consecutive patients with a spinal cord injury, without bone injury, and ≥ 50 years of age were included. Multivariate regression analysis was used to compare the operative time between patients in the TP and HA group, adjusting for known factors that can influence surgical and postoperative outcomes. Propensity score matching was used to confirm the robustness of the primary outcome. The cumulative incidence of postoperative complications over 1-year after surgery was also compared.

**Results:**

Of the 164 patients forming our study group, TP spacers were used in 62 and HA in 102. Operative time was significantly shorter for the TP (128 min) than HA (158 min) group (*p* < 0.001). Both multivariate and propensity score matching analyses confirmed a significant reduction in operative time for the TP, compared to HA, group (regression coefficient, − 30 min and − 38 min, *p* < 0.001 and *p* < 0.001, respectively). There was no significant difference in the cumulative incidence of postoperative complications.

**Conclusions:**

The use of TP spacers reduced the operative time for cervical open-door laminoplasty by about 30 min, compared to the use of HA spacers, with no difference in the rate of postoperative complications.

## Background

The main application of cervical laminoplasty is the treatment of degenerative spinal disease, such as cervical spondylitis myelopathy (CSM), and spinal trauma, such as spinal cord injury without bone injury (SCIBI), also known as central cord syndrome. Cervical canal stenosis is a major cause of spinal cord injury in these cases, with the stenosis being congenital in nature or resulting from degenerative changes. Cervical laminoplasty provides a posterior approach to decompress the spinal cord via the opening of the laminae at multiple levels, with reports of good clinical results [1, 2]. There are two types of cervical laminoplasty, namely, door and double door [3], with no significant difference in clinical and radiological results having been reported between the two approaches [4, 5].

In the original open-door laminoplasty technique, the opened laminae were held with sutures, placed between the laminae, and by the muscles adjacent to the facet joints [6]. However, postoperative laminae reclosure was a major complication of this approach [7, 8]. Hence, several modifications have been developed to address this limitation of open-door laminoplasty, including the use of suture anchors, hydroxyapatite block (HA) spacers and titanium plate (TP) spacers [9, 10], with recent meta-analyses indicating similar good clinical results among these procedures [11, 12]. However, it is important to note that an increase in operative time during spinal surgery increases the incidence of postoperative complications, such as urinary tract infection, prolonged intubation, and vision loss [13–15]. These complications related to a prolonged operative time would be particularly critical for frail elderly individuals who are at the highest risk for CSM and SCIBI. Therefore, spinal procedures that can shorten the operative time would be specifically important for this clinical population to reduce the risk of postoperative complications.

The selection of spacers may be an important consideration with respect to operative time for cervical laminoplasty. Specifically, HA spacers require the creation of bone burrs to pass the sutures, whereas TP spacers require only the creation of small holes for screws. As such, TP spacers might be easier to use than HA spacers, with the possibility of reducing operative time for cervical laminoplasty. However, the operative time has not been specifically compared between the use of TP and HA spacers during cervical laminoplasty. Our study specifically addressed this issue, with the aim being to compare operative time for cervical laminoplasty using TP and HA spacers, with multivariate analyses applied to adjust for confounding factors. We hypothesized, *a priori*, that operative time would be shorter when using TP than HA spacers.

## Methods

### Study design and setting

We conducted a retrospective observational study of patients who underwent surgical treatment for SCIBI at two level one trauma centers in Japan, between January 2009 and December 2017. Patients were retrospectively identified via a search of the surgical databases at our two affiliated hospitals. Demographic information, treatment, and postoperative clinical course were extracted from each patient’s electronic medical record.

Our study protocol was approved by the institutional review board of each hospital.

### Patients

The inclusion criteria were as follows: cervical SCIBI, with an American Spinal cord Injury Association impairment scale (AIS) classification D; surgical treatment for the SCIBI performed between January 2009 and December 2017; patient age ≥ 50 years; and treated using an open door laminoplasty, performed within 1 week of the injury. Patients with a concomitant head injury (with an abbreviated injury scale grade ≥ 2), multiple trauma (with an injury severity score ≥ 16), and/or a past history of any SCI were excluded.

### Description of surgical procedure

**Fig. 1:**
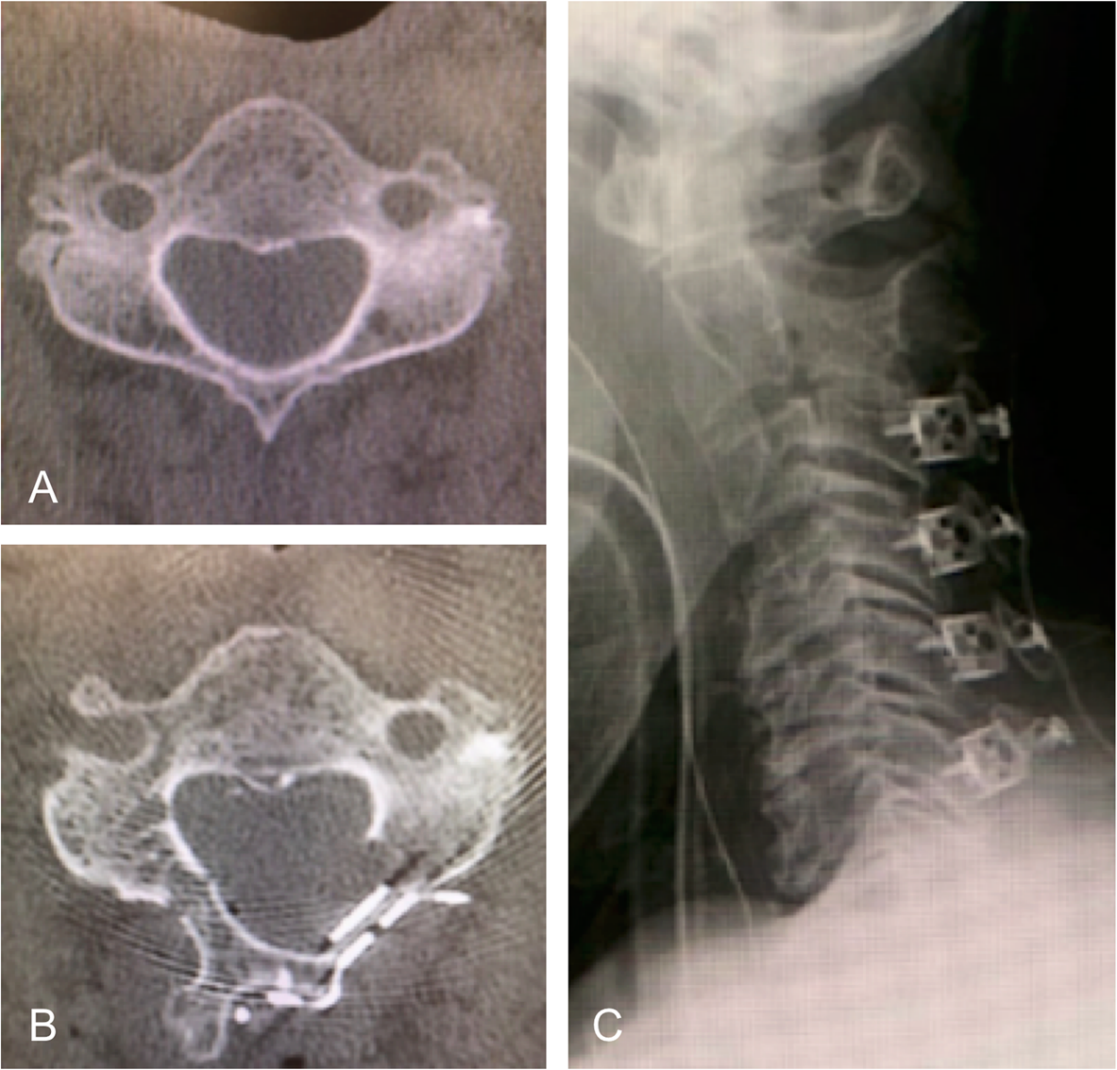
Example of a case of open door laminoplasty using titanium plate spacers. **a** Preoperative computed tomography image. **b** Postoperative computed tomography image. **c** Postoperative radiograph

**Fig. 2:**
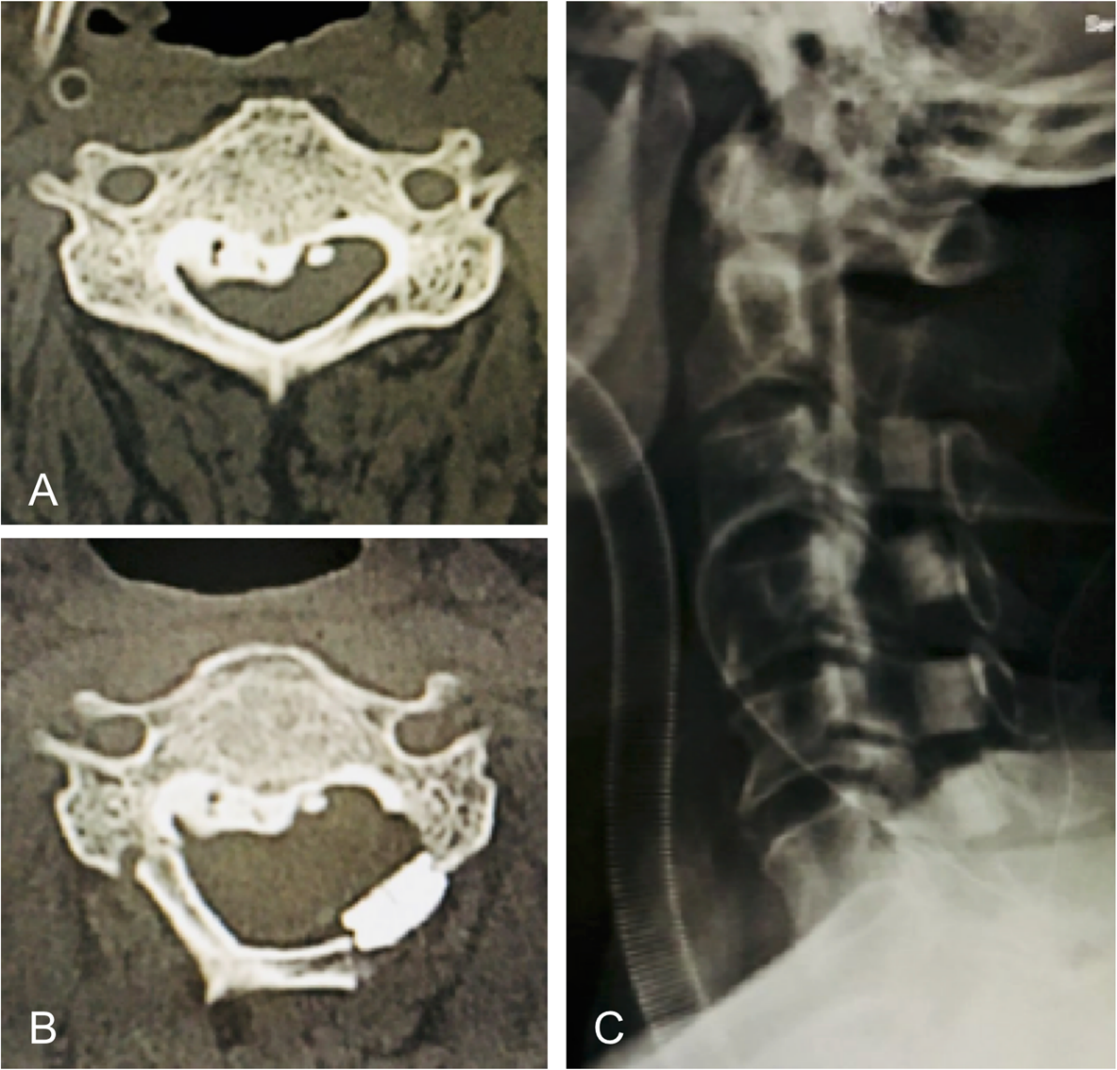
Example of a case of open door laminoplasty using hydroxyapatite block spacers. **a** Preoperative computed tomography image. **b** Postoperative computed tomography image. **c P**ostoperative radiograph

All procedures were performed with the patient in the prone position, with a Mayfield head fixator applied, under general anesthesia. A standard posterior midline approach was used to expose the target cervical laminae. Using a high-speed drill bar and Kerrison punch, bilateral gutters were created at the junction of the target laminae and the medial end of the lateral masses. On the left side, both cortices were completely cut, creating an *opened* side, while on the right side, the anterior cortex was cut to be used as a hinge. The flap of the lamina was lifted and kept open by inserting one either a TP or HA spacer, with the appropriate size selected for each patient. The TP spacer (Basket plate, HOYA Technosurgical Co., Japan) was fixed by two screws (Fig. 1) and has a basket in the middle of the plate that is packed with a mixture of HA granules and collagen (Refitsoft HA tissue, HOYA Technosurgical Co., Japan) to induce bone induction. The HA spacer (APACERAM, HOYA Technosurgical Co., Japan) was fixed using sutures passed through bone burrs created in the laminae and the medial end of the lateral mass (Fig. 2). Following surgery, all patients were permitted to move their neck immediately, with rehabilitation initiated on postoperative day 1.

### Main exposure

For analysis, patients were classified into two groups, based on the type of spacer used, TP or HA. Before mid-2016, only HA spacers were used; thereafter, the type of spacer selected was based on each surgeon’s preference.

### Outcomes

The primary outcome was the operative time, which was defined as the time from the first incision to closure. The secondary outcomes were the volume of intraoperative bleeding, the prevalence of the postoperative cervical kyphosis measured by radiography and the postoperative diameter of the cervical canal in sagittal plane measured by radiography [16] and the cumulative incidence of complications, including the need for reoperation, deep surgical site infection, postoperative C5 palsy, and failure of the spacers within one year after surgery.

### Variables

The following patient demographics and surgery-related variables were included as covariates: sex, age, body mass index (BMI), American Society of Anesthesiologists (ASA) physical status (dichotomized as a score < 3 or ≥ 3), ossification of the posterior longitudinal ligament (OPLL), diabetes mellitus, antiplatelet, or anticoagulant therapy, surgeon’s experience (consultant or surgical trainee), time of the surgery (day or night), number of laminae decompressed, preoperative diameter of the cervical canal by computed tomography (CT) imaging [16], inclusion of the dens in the laminoplasty, and preoperative analyses (hemoglobin and platelet count, estimated glomerular filtration rate, prothrombin time-international normalized ratio (PT-INR), and activated partial thromboplastin time.

### Statistical analysis

Demographic data were compared between the two groups (TP and HA), using a *t* test for continuous variables and the chi-squared test for categorical variables. The primary and secondary outcomes were compared between the two groups using a *t* test or chi-squared test as appropriate for the data type and distribution. Multivariate regression analysis was used to evaluate the effect of using a TP spacer, in an open door laminoplasty procedure, on the primary outcome, adjusted for potential confounding factors, including age, sex, OPLL, number of decompressed laminae, and laminoplasty of the dens, selected based on a previous study [17] and the surgeon’s experience. The regression coefficient (RC), 95% confidence intervals (CI), and *p* value were also determined.

We performed two sensitivity analyses, using propensity scores (PS), to investigate the robustness of the primary analysis: PS-controlled multiple regression analysis and a PS-matched analysis [18]. The PS was generated for each patient from the multiple logistic regression model, calculating the probability of a TP spacer being selected based on preoperative variables. For the PS-matched analysis, each case in the TP group was matched to a case in the HA group, based on the closest PS (within 20%). The RC, 95% CI, and *p* value were calculated for all estimates. All analyses were performed using STATA (version 15.0; Stata Corp. LLC, College Station, TX, USA). A two-sided *p* value < .05 was considered statistically significant.

## Results

During the study period, a total of 164 patients were eligible for participation, 62 treated using TP spacers and 102 using HA spacers, with a combination of TP and HA spacers not used in any patient. The general demographic characteristics of the 164 patients were presented in Table 1, stratified according to the type of spacer used. There were significant differences between the two groups with regard to the proportion of diabetes mellitus, surgeon’s experience, number of decompressed laminae, and the preoperative PT-INR.

**Table 1:** Demographic characteristics of the patients

Variables	TP group (*n* = 62)	HA group (*n* = 102)	*p* value
Sex, male, *n* (%)	47 (76)	83 (81)	0.39
Age, mean (SD), years	70 (8.0)	68 (9.3)	0.06
BMI, mean (SD), kg/m^2^	24 (3.7)	23 (3.4)	0.60
ASA physical status (≥ 3), *n* (%)	9 (15)	19 (19)	0.50
OPLL, *n* (%)	17 (27)	27 (26)	0.89
Diabetes mellitus, *n* (%)	23 (37)	13 (13)	< 0.001
Antiplatelet or anticoagulant medicine, *n* (%)	6 (9.7)	13 (13)	0.55
Level of operator (consultant), *n* (%)	17 (27)	51(51)	0.003
Timing of operation (day time), *n* (%)	34 (55)	58 (57)	0.80
Number of DL, mean (SD)	4.3 (0.60)	4.0 (0.64)	0.03
Preoperative diameter of cervical canal, mean (SD), mm	12 (1.2)	12 (1.7)	0.11
Laminoplasty of dens, *n* (%)	13 (21)	37 (36)	0.04
Hemoglobin, mean (SD), g/dl	13 (2.0)	14 (1.7)	0.42
Platelet, mean (SD), 10^4^/μl	21 (7.0)	21 (17)	0.99
eGFR, mean (SD), ml/min/1.73 m^2^	69 (25)	71 (20)	0.51
PT-INR, mean (SD)	0.98 (0.13)	1.1 (0.20)	0.01
APTT, mean (SD), s	29 (5.5)	27 (4.2)	0.07

Abbreviations: *TP* titanium plate, *HA* hydroxyapatite block, *SD* standard deviation, *BMI* body mass index, *ASA* American Society of Anestheologists, *OPLL* ossification of posterior longitudinal ligament, *DL* decompressed lamina, *eGFR* estimated glomerular filtration rate, *PT-INR* prothrombin time-international normalized ratio, *APTT* activated partial thromboplastin time

**Fig. 3:**
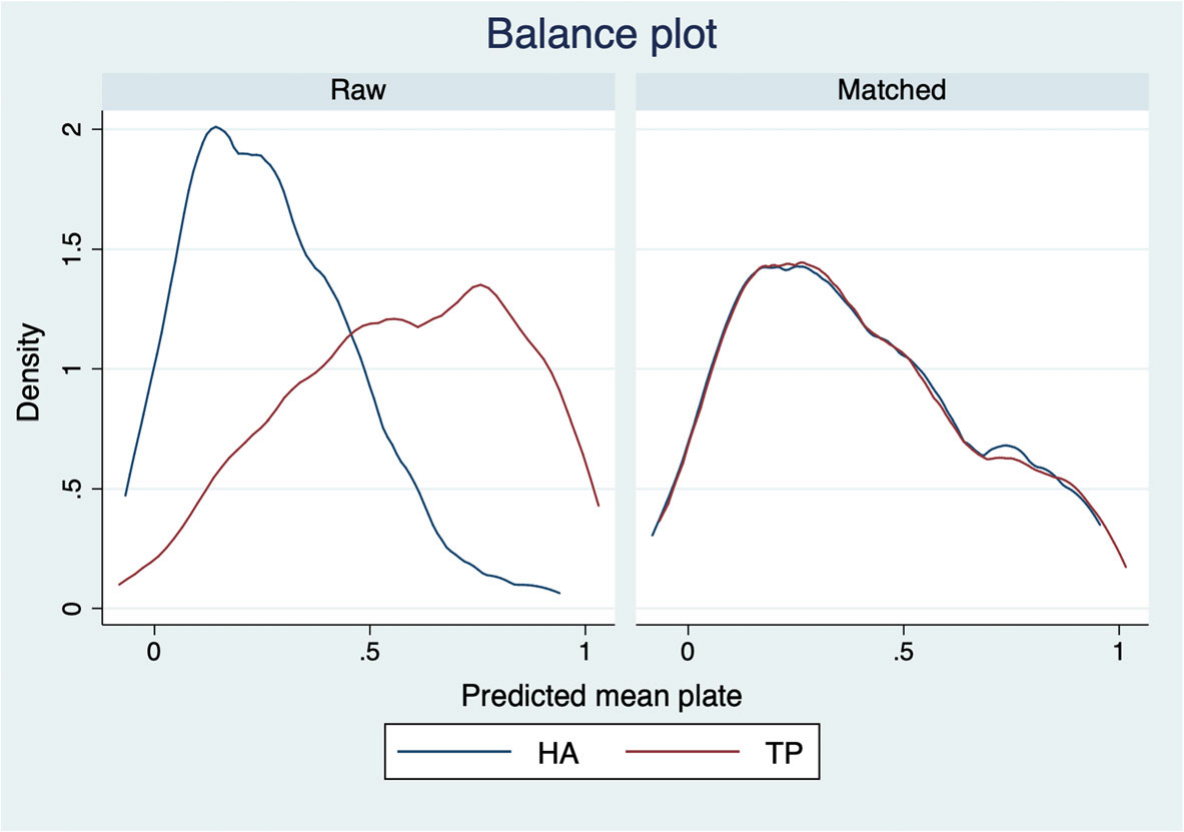
Balance plot between the two groups before and after propensity score matching. Abbreviations: TP: titanium plate, HA: hydroxyapatite block

On univariate analyses for the primary outcome, operative time was significantly shorter for the TP than the HA group: 128 min versus and 158 min, respectively; *p* < 0.001; Table 2. The shorter operative time for the TP group was confirmed on multivariate analysis: RC, − 30 min; 95% CI, − 42 to − 17 min; *p* < 0.001, Table 3. In the sensitivity analyses, the multiple logistic regression to derive the PS had a C-statistic of 0.82, and the balance plot confirmed that the TP and HA groups were well-balanced after matching (Fig. 3). Both PS analyses confirmed a significantly shorter operative time for the TP than HA group (Table 4). With regard to the secondary outcomes, the volume of intraoperative bleeding was significantly greater in the HA (230 ml) than in the TP (146 ml) group (*p* < 0.001; Table 2). In addition, although the difference was small, the postoperative diameter of the cervical canal was significantly greater in the TP (18 mm) than the HA (17 mm) group (*p* < 0.001; Table 2). There was no significant difference in the prevalence of postoperative cervical kyphosis nor in the cumulative incidence of postoperative complications within 1 year after surgery between the two groups (Table 2).

**Table 2:** Univariate analysis of primary and secondary outcome measures

Variables	TP group (*n* = 62)	HA group (*n* = 102)	*p* value
Operation time, mean (SD), min	128 (32)	158 (44)	< 0.001
Intraoperative bleeding, mean (SD), ml	146 (13)	230 (20)	0.003
Postoperative cervical kyphosis, *n* (%)	6 (9.7)	13 (13)	0.55
Postoperative diameter of cervical canal, mean (SD), mm	18 (2.2)	17 (2.2)	< 0.001
Complication required another surgery, *n* (%)	2 (3.2)	5 (4.9)	0.61
Deep surgical site infection, *n* (%)	1 (1.6)	3 (2.9)	0.59
C5 palsy, *n* (%)	0 (0)	1 (1.0)	0.43
Failure of spacers, *n* (%)	0 (0)	0 (0)	1.0

Abbreviations: *TP* titanium plate, *HA* hydroxyapatite block

**Table 3:** Multiple regression analysis of the primary outcome

Adjusted variables	Coefficient	95% confidence interval	*p* value
Using TP spacers	− 30	− 42 to − 17	< 0.001
Using HA spacers	reference		
Age	0.05	− 0.63–0.73	0.89
Sex (male)	11	− 3.7–25	0.14
OPLL	3.4	− 10–17	0.63
Number of DL	16	6.1–26	0.002
Laminoplasty of dens	18	4.0–32	0.01

Abbreviations: *TP* titanium plate, *HA* hydroxyapatite block, *OPLL* ossification of posterior longitudinal ligament, *DL* decompressed lamina

**Table 4:** Regression coefficient of using TP spacers

Model	Coefficient	95% confidence interval	*p* value
Crude	− 30	− 30 to − 6.4	< 0.001
Multiple regression analysis without using PS	− 30	− 42 to − 17	< 0.001
PS controlled multiple regression analysis	− 35	− 51 to − 20	< 0.001
PS matching	− 38	− 52 to − 23	< 0.001

Abbreviations: *TP* titanium plate, *PS* propensity score

The propensity score was calculated by a logistic regression model using the following variables: sex, age, body mass index, American Society of Anestheologists physical status, ossification of posterior longitudinal ligament, diabetes mellitus, use of antiplatelet or anticoagulant medicine, level of operator, number of decompressed laminae, laminoplasty of dens, hemoglobin, platelet, estimated glomerular filtration rate, prothrombin time-international normalized ratio, activated partial thromboplastin time

## Discussion

Our retrospective observational study indicated that the use of TP spacers during open-door laminoplasty can reduce operative time by about 30 min, compared to the use of HA spacers. The use of TP spacers also decreased the volume of intra-operative bleeding, with no difference in the cumulative incidence of postoperative complications between the two procedures.

To our knowledge, this is the first study to have directly compared the operative time between the use of TP and HA spacers in open-door laminoplasty. Previous studies have reported an operative time of 112–160 min for HA spacers [19–21], compared to 90–181 min for TP spacers [22–26]. However, as these were single-arm descriptive studies of either procedure without direct comparison, the benefit of TP spacers on operative time could not be confirmed. TP spacers are thought to be easier to use than HA spaces as only screws are needed to fix the plate. As such, orthopedic surgeons may select to use TP spacers more frequently than HA spacers for patients for whom a shorter operative time would be preferable, such as those with OPLL, older patients, and those requiring a large number of laminae to be decompressed. However, these indications for TP over HA have not previously been confirmed in a head-to-head comparison and, thus, the indications might be influenced by systematic error [27]. Our study provides this confirmation, with our use of multivariate linear regression to adjust for confounding factors on the primary outcome, operative time. We do note that the result of our multivariate analysis was not different from the results of our univariate analyses.

A possible limitation of multivariate regression analysis is *overfitting* of the model which may occur when too many confounding factors are adjusted for [28]. To cope with this problem, we selected only five confounding factors to include in the model. These were considered to have a potentially important influence on the primary outcome, based on previous research. However, this may have resulted in residual confounding effects on the primary outcome. To evaluate this possibility, we performed PS sensitivity analyses, with the PS based on the probability of a patient being assigned to receive either TP or HA spacers. After PS matching, both the TP and HA groups were well-balanced (Fig. 3), allowing for a direct comparison of the effect of TP and HA spacers on operative time. Results of both PS-based sensitivity analyses were nearly the same, with the coefficient of between-group difference being larger than the crude and multivariate coefficient (Table 4). Therefore, the PS-based analyses further adjusted for residual confounding effects on the primary outcome. Based on our findings and on those from previous studies, we propose that the use of TP spacers, over HA spacers, will reduce the operative time of an open door laminoplasty.

The cost is different for TP and HP spacers. In Japan, TP spacers cost about $1500 per lamina, compared to about $350 for HA spacers. Therefore, for a case requiring laminae decompression at four levels, the use of TP spacers would cost $4600 more than the use of HA spacers. This higher cost for TP spacers would not be equivalent to the decrease in cost associated with a 30-min shortening in operative time, when we consider the cost of anesthesia and labor cost of the surgical staff. Our findings also demonstrated that the rate of postoperative complications was not different between the two groups, which was consistent with the findings of a recent meta-analysis that reported no difference in neurological outcomes between the use of TP spacers and other procedures [11]. However, we do need to consider that the prolonged operative time in a prone position, as required for cervical laminoplasty, would increase the risk of postoperative complications. Therefore, a shorter operative time is desirable, particularly for older patients [29].

The prevalence of cervical laminoplasty has increased with the general aging of the population, with aging being an important risk factor for CSM and SCIBI [30, 31]. The use of TP spacers for cervical laminoplasty has been questioned from a cost-effectiveness perspective. However, our results indicate that the use of TP spacers would be recommended in cases where the operative time might have an important influence on the postoperative prognosis, such as for patients with multiple trauma, significant anemia, or very poor lung function.

The limitations of our study need to be acknowledged in the interpretation of our findings for practice. First, the target population in our study was patients with SCIBI, with an AIS-D classification; as such, the application of our findings to patients with other spinal diseases or traumas is limited. However, our findings would be useful to inform the selection of spacers for patients with CSM, as the characteristics of these patients is generally similar to those of our study population. Second, because of the retrospective design of our study and the fact the spacer type was selected by the individual surgeon, based on preference, which might have introduced a confounding effect, a causal relationship between the use of TP spacers and a shorter operative time cannot be confirmed. Although we used a multivariate analysis and included PS-based analyses, including matching for the level of a surgeon’s experience, the residual effects of confounding factors cannot be denied. Third, we only considered the effects of the TP and HA spacers on operative time. However, other potential confounding factors should be considered, such as the required learning curve and continued technical improvements in operative procedures, which would influence the selection of the type of spacer, as well as the operative time itself. Performing a randomized control trial would be necessary to solve these limitations. However, considering important outcomes, such as neurological prognosis, is not expected to be influenced by the selection of spacers [11], the relevance of conducting such a trial would be very low. Therefore, we believe that our results would be sufficient to inform practice.

## Conclusions

The use of TP spacers for open-door cervical laminoplasty was associated with a reduction in the operative time of about 30 min, compared to the use of HA spacers, without increasing postoperative complications.

## Data Availability

The datasets used and/or analyzed during the current study are available from the corresponding author on reasonable request.

## Abbreviations

AIS: American Spinal cord Injury Association impairment scale
ASA: American Society of Anesthesiologists
BMI: Body mass index
CI: Confidence intervals
CSM: Cervical spondylitis myelopathy
CT: Computed tomography
HA: Hydroxyapatite block
OPLL: Ossification of the posterior longitudinal ligament
PS: Propensity score
PT-INR: Prothrombin time-international normalized ratio
RC: Regression coefficient
SCIBI: Spinal cord injury without bone injury
TP: Titanium plate
